# The effectiveness of cyclosporine A for patients with steroid-resistant nephrotic syndrome: A protocol for systematic review and meta-analysis

**DOI:** 10.1097/MD.0000000000028186

**Published:** 2021-12-10

**Authors:** Juan Lv, Shizhi Luo, Yunxia Zhang, Enlai Dai

**Affiliations:** a College of Integrated Traditional Chinese and Western Medicine, Gansu University of Traditional Chinese Medicine, Gansu, China; b Department of Emergency, Gansu Hospital of Traditional Chinese Medicine, Gansu, China; c Department of Song's Orthopedics, Affiliated Hospital of Gansu University of Traditional Chinese Medicine, Gansu, China; d Department of Neurology, Gansu Hospital of Traditional Chinese Medicine, Gansu, China; e Integrated Traditional Chinese and Western Medicine College, Gansu University of Traditional Chinese Medicine, Gansu, China.

**Keywords:** Cyclosporine A, end-stage renal disease, meta-analysis, steroid-resistant nephrotic syndrome

## Abstract

**Background:**

The purpose of this study is to determine the efficacy and safety of Cyclosporine A (CsA) for patients with steroid-resistant nephrotic syndrome (SRNS).

**Methods:**

This study will be designed following the guidelines of the Preferred Reporting Items for Systematic Reviews and Meta-Analyses Protocols statement guidelines. Studies are identified through systematic searches in November 2021 with no restrictions on date and time, and publication status using the following bibliographic databases: Embase, Medline, PubMed, Web of Science, Science Direct, and the Cochrane Library. The risk of bias of included studies is estimated by taking into consideration the characteristics including random sequence generation, allocation concealment, blinding of patients, blinding of outcome assessment, completeness of outcome data, selective reporting, and other bias by Cochrane Collaboration's tool. Data synthesis and analyses are performed using Stata version 10.0 software.

**Results:**

The results of this systematic review and meta-analysis will be published in a peer-reviewed journal.

**Conclusion:**

CsA may be an effective and safe therapy for SRNS. However, additional randomized controlled studies are needed to thoroughly assess the role of CsA in the treatment of SRNS.

**Open Science Framework registration number:**

10.17605/OSF.IO/P6YB9

## 1 Introduction

Nephrotic syndrome (NS), characterized by hypoalbuminemia, massive proteinuria, peripheral edema, and hyperlipidemia, is a major cause of end-stage renal disease (ESRD), and related damage of the glomerular filtration barrier.^[1–3]^ Based on the response to steroid therapy, NS is classified as steroid-sensitive nephrotic syndrome (approximately 50% of steroid-sensitive nephrotic syndrome patients develop frequently relapsing nephrotic syndrome or steroid-dependent nephrotic syndrome), or steroid-resistant nephrotic syndrome (SRNS).^[4]^ Patients who do not enter remission after administration of daily prednisolone for 4 weeks are regarded as SRNS.^[5,6]^ Steroid resistance most often occurs during initial treatment with prednisolone (initial resistance), but can also occur during treatment for a relapse, in a patient who had previously responded to treatment with steroids or with a second-line drug (late resistance).^[7,8]^ SRNS is regarded as one of the most common causes of the development of ESRD in children.^[9]^

The current therapeutic options for SRNS are often ineffective, it frequently progresses to a loss of kidney function, and treatment is often complicated by significant toxicity associated morbidities, mortality, and cost. Efforts to improve the outcome of patients with SRNS have resulted in the emergence of various steroid sparing agents with considerations for sustained remission and reduced side effects. These include alkylating agents such as cyclophosphamide, calcineurin inhibitors such as cyclosporine and tacrolimus, mycophenolate mofetil, a T and B cell proliferation inhibitor and rituximab, a monoclonal antibody generally reserved for multitherapy resistant cases.^[10–12]^ These have been used singly or in combination with wide variations in practice among pediatric nephrologists worldwide and consequently, varying outcomes. In the current study, we performed a protocol for systematic review and meta-analysis to assess the safety and efficacy of Cyclosporine A (CsA) in the treatment of patients with SRNS.

## 2 Methods

This meta-analysis was registered at Open Science Framework registries (registration number: 10.17605/OSF.IO/P6YB9) and was conducted in accordance with the guidelines of the Preferred Reporting Items for Systematic Reviews and Meta-Analyses Protocols statement guidelines.^[13]^ Ethics application was not required as this study is based on published trials.

### 2.1 Search strategy

Studies were identified through systematic searches in November 2021 with no restrictions on date and time, and publication status using the following bibliographic databases: Embase, Medline, PubMed, Web of Science, Science Direct, and the Cochrane Library. The following subject heading terms or keywords were used in our search: cyclosporine, nephrotic syndrome, glomerulonephritis membranoproliferative, focal segment glomerulosclerosis, and minimal change nephrotic syndrome. Boolean operators such as “AND” and “OR” were used to combine search terms. The reference lists of the included studies were also checked for additional studies that were not identified with the database search.

### 2.2 Inclusion and exclusion criteria

In this study, the inclusion criteria were as follows: investigation type: randomized controlled trials; object of the study: patients were diagnosed with NS and the NS was resistant to the steroid treatment; type of interventions: treatment groups received CsA, the controls should have been treated with another immunotherapy or placebo.

### 2.3 Exclusion criteria

Exclusion criteria for the study were as follows: reviews, case reports, letters, systematic reviews, and meta-analysis; patients with NS were sensitive to steroid or dependent to steroid; studies that do not contain different therapeutic regimens; the diagnostic criteria were not clear.

### 2.4 Data extraction

Two independent authors will extract the below descriptive information from the included articles: demographic information of patients, such as average age, number of patients, sex ratio, and body mass index; study characteristics, such as authors, year of publication, study language, study design, and the average follow-up period; details of interventions and outcome measures. If the data cannot be directly extracted or is missing, we will contact the relevant author to ensure that the information is complete. Otherwise, we will calculate them with the guideline of Cochrane Handbook for Systematic Reviews of Interventions 5.1.0.

### 2.5 Quality evaluation

The risk of bias assessment of the included studies was performed by 2 authors independently using the Cochrane Collaborations risk of bias tool. This tool included 7 aspects that were sequence generation (selection bias), allocation sequence concealment (selection bias), blinding of participants and personnel (performance bias), blinding of outcome assessment (detection bias), incomplete outcome data (attrition bias), selective outcome reporting (reporting bias), and other bias (baseline balance and fund). Additionally, each of the aspects was ranked low risk of bias, high risk of bias, and unclear risk of bias.

### 2.6 Statistical analysis

We performed the meta-analysis by Stata version 10.0 software and calculated the statistics using the inverse variance statistical method. Continuous variables were expressed as the weighted mean difference or standardized mean difference and 95% confidence interval (CI). Weighted mean difference was used when data were measured in the same scale and standardized mean difference was used if data were measured using different scales. Heterogeneity among the studies was quantified with the I^2^ statistic. If I^2^ > 50% or *P* < .1, a random-effect model was used to decrease heterogeneity, and the subgroup and sensitivity analysis were performed to explore the sources of heterogeneity; Otherwise, heterogeneity was negligible and a fixed-effect model was used. To evaluate publication bias, we perform a funnel plot if the number of included studies is sufficient (>10 articles). A symmetrical funnel plot indicates no possibility of publication bias, while an asymmetrical funnel plot indicates a high possibility of publication bias. If we identify publication bias through analysis of the funnel plot, we may discuss possible reasons such as small-study effects.

## 3 Discussion

Patients with SRNS are at risk of progressing to ESRD. Renal histology in most patients shows presence of focal segmental glomerulosclerosis, minimal change disease, and (rarely) mesangioproliferative glomerulonephritis.^[14]^ A third of patients with SRNS show mutations in one of the key podocyte genes. The remaining cases of SRNS are probably caused by an undefined circulating factor.

The biggest challenge, as we see it, is to define the presumed circulating factors that cause the disease in most patients with SRNS. The nature of this factor has been investigated for several decades without success. The precise effect of this circulating factor on podocyte biology also needs to be clarified.^[15]^ We suspect that the circulating factor is in some way related to the immune system, since all currently effective treatments are with immunosuppressive medications. The purpose of this study is to determine the efficacy and safety of CsA for patients with SRNS. However, the number of genes causing SRNS will undoubtedly continue to rise until all have been found. Patients with SRNS who have an underlying genetic disorder are unlikely to respond to medications and might be future candidates for genetic therapy, including genomic editing.^[16,17]^

## Author contributions

**Conceptualization:** Shizhi Luo.

**Data curation:** Juan Lv and Shizhi Luo.

**Funding acquisition:** Enlai Dai.

**Investigation:** Yunxia Zhang.

**Methodology:** Juan Lv, Yunxia Zhang.

**Writing – original draft:** Juan Lv.

**Writing – review & editing:** Enlai Dai.
